# Recent Advances in Obesity: Genetics and Beyond

**DOI:** 10.5402/2012/536905

**Published:** 2012-03-05

**Authors:** Wai W. Cheung, Peizhong Mao

**Affiliations:** ^1^Division of Pediatric Nephrology, Department of Pediatrics, University of California, San Diego, La Jolla, CA 92093, USA; ^2^Division of Neuroscience, Oregon National Primate Research Center, Department of Public Health & Preventive Medicine, Oregon Health & Science University, Portland, OR 97239, USA

## Abstract

The prevalence of obesity, which is a heritable trait that arises from the interactions of multiple genes and lifestyle factors, continues to increase worldwide, causing serious health problems and imposing a substantial economic burden on societies. For the past several years, various genetic epidemiological approaches have been utilized to identify genetic loci for obesity. Recent evidence suggests that development of obesity involves hormones and neurotransmitters (such as leptin, cocaine- and amphetamine-regulated transcript (CART), and ghrelin) that regulate appetite and energy expenditure. These hormones act on specific centers in the brain that regulate the sensations of satiety. Mutations in these hormones or their receptors can lead to obesity. Aberrant circadian rhythms and biochemical pathways in peripheral organs or tissues have also been implicated in the pathology of obesity. More interestingly, increasing evidence indicates a potential relation between obesity and central nervous system disorders (such as cognitive deficits). This paper discusses recent advances in the field of genetics of obesity with an emphasis on several established loci that influence obesity. These recently identified loci may hold the promise to substantially improve our insights into the pathophysiology of obesity and open up new therapeutic strategies to combat growing obesity epidemic facing the human population today.

## 1. Introduction

Throughout history, science has invested enormous time and effort in the search to understand the physiological basis of obesity. Crucial to this research are the inquiry of how does our body control ingestion, digestion, absorption, and metabolism and how nutrients are distributed among various tissues, organs, and systems [1, 2]. Overweight and obesity, defined as body mass index (BMI, the weight in kilograms divided by the square of the height in meters) >25 and >30, respectively, are associated with premature death through increased risk of many chronic diseases, including type 2 diabetes, cardiovascular disease, and cancer [3–5]. Obesity is a major international public health threat and economic burden. Over the last 3 decades, the prevalence of overweight and obesity has increased rapidly. The latest World Health Organization estimates that 1.6 billion adults (aged 15 years and above) were overweight and 400 million were obese in 2005. These figures are predicted to rise to 2.3 billion overweight and over 700 million obese adults by 2015 [6].

Obesity results when body fat accumulates over time as a result of chronic energy imbalance (calories consumed exceed calories expended). In recent decades, obesity has reached epidemic proportions in many populations whose environments offer an abundance of calorie-rich foods and few opportunities for physical activities. Obesity is caused by a complex interaction of the environment, the genetic predisposition, and human behavior, but the relative contribution of these factors is still poorly understood. Environmental factors are major contributors to the obesity epidemic. In addition to environmental factors, there is genetic predisposition to obesity. Although changes in genetic makeup of populations may not be fully responsible for this rapid rise in obesity, genetic predisposition does play a vital role in the development of obesity. Genetic factors are estimated to account for >40% of the population variation in BMI [7, 8]. Thus, genes influence how our bodies capture, store, and release energy from food.

How might genes contribute to obesity? Currently, there are quite a few theories that (the thrifty gene hypothesis, the fetal programming hypothesis, the predation release hypothesis, the sedentary lifestyle hypothesis, the ethnic shift hypothesis, the increased reproductive fitness hypothesis, the assortative mating hypothesis, and the complex hypothesis) intend to explain the genetic basis of human obesity [9, 10]. However, an acceptable consensus in the field is still lacking, probably due to the fact that complex genetic interactions influence the development of obesity. One of the intriguing explanations for the rapid rise in obesity is the mismatch between today's environment and “energy thrifty genes” that multiplied in the past under different environmental conditions when food sources were rather scarce. In other words, according to the “thrifty genotype” hypothesis, environments in which food is plentiful year around are now challenging the same genes that helped our ancestors survive occasional famines. It has been argued that the thrifty genotype is just part of a wider spectrum of ways in which genes can favor fat accumulation in a given environment. These ways include the desire to overeat; the tendency to be sedentary; a diminished ability to utilize dietary fats as fuel; an enlarged, easily stimulated capacity to store body fat [11, 12]. The variation in how people respond to the same environmental conditions is an additional indication that genes play an important role in the development of obesity [13]. This is also consistent with the notion that obesity results from genetic variation interacting with shifting environmental conditions. Recent advances in genetic epidemiological approaches have identify several genetic loci for obesity. This review will focus on several established loci that influence obesity. Identifying the genetic factors underlying the heritable risk of obesity will contribute to our basic knowledge of the biology of energy balance, and might even highlight molecules and signaling pathways that can be targeted for therapeutic intervention.

## 2. Approaches to Identify Human Obesity Genes

The genetic contribution to common obesity has been established through family, twin, and adoption studies. Twin studies have shown that genetic factors may contribute to >40% of the variation in BMI while lower heritability has been shown in families (>20%) and adoption (>20%), respectively [7, 8, 14, 15]. Yet, despite a relatively high heritability, the search for obesity susceptibility genes has been an arduous task. Progress until recently has been slow and success limited. Although recent success of genome-wide association studies has drawn a lot of attention, gene identification for the last 15 years has been based on two broad genetic epidemiological approaches, that is, candidate gene and genome-wide linkage methods.

### 2.1. Candidate Gene Approach

The candidate gene approach is a hypothesis-driven approach that relies on current understanding on the biology and pathophysiology of the disease. The candidacy of a gene for obesity is based on the following resources: animal models using gene knockout and transgenic approaches; cellular model systems showing their role in metabolic pathways involved in glucose metabolism; linkage and positional cloning studies using extreme cases. The concept of this approach is to identify an association between a variant or mutation within or near the candidate gene and a trait of interest (such as obesity). Candidate gene approach needs to be on a large scale and well powered in order to detect the expected small effects of genetic variants involved in common traits and disease [16, 17]. For example, neuropeptide cocaine- and amphetamine-regulated transcript (CART) was reported as an endogenous satiety factor [18]. Mutational screening of the CART gene in obese children has identified a mutation (Leu34Phe) associated with reduced resting energy expenditure and obesity phenotype [19]. A recent study shows that a small group of adolescents with the Leu34Phe mutation in the CART gene exhibit higher anxiety and depression scores than control subjects and develop severe early-onset obesity [20]. Results suggest a possible relationship between prevalence of obesity and severity of mental disorders, in which CART may play a unique role [21].

Genotyping costs have come down substantially, and publicly available datasets, such as dbSNP and the International HapMap, have provided deeper insight into genetic variation in genes. This knowledge has led to more comprehensive studies that systematically examine the association of all common variation in a gene of interest by means of carefully selected tagSNPs and their haplotypes under the assumption that a causal variant would be in high linkage disequilibrium with one of the tagSNPs or at least captured by the haplotypes [22]. Perhaps the most important finding in hunting obesity gene occurred in 1994, when Zhang and coworkers demonstrated for the first time that an adipose-derived hormone, leptin, plays a key role in regulating intake and energy expenditure, including appetite and metabolism [23, 24]. This discovery represented a huge step forward in the study of obesity. Mutations in leptin and leptin receptor genes have been associated with mild to extreme obesity phenotype in human population [25–28].

Since 1994, the number of proposed obesity susceptibility genes has grown steadily. The latest update of the Human Obesity Gene Map reported 127 candidate genes for obesity-related traits [29]. Results of large-scale studies suggest that obesity is strongly associated with genetic variants in the melanocortin-4 receptor (MC4R) gene, adrenergic *β*3 receptor (ADRB3) gene, prohormone convertase 1 (PCSK1) gene, brain-derived neurotrophic factor (BDNF) gene, and endocannabinoid receptor 1 (CNR1) gene (Table 1). Relevance of those putative genes confer to the development of obesity has been recently summarized [9, 22]. Hypothalamic leptin-melanocortin system is critical for energy balance in humans, because disruption of this network causes the most severe obesity phenotypes [30–32].

Notably, leptin administration is effective in treating obesity. Long-term therapy with subcutaneous injections of recombinant leptin provided sustained beneficial effects on lowering fat mass, hyperinsulinemia, and hyperlipidemia in human obese subjects [33]. These results suggest an important role of leptin in the regulation of the human hypothalamic-pituitary-thyroid axis. It is important to highlight that this strategy is effective for patients with leptin deficiency, but not necessarily applicable as a treatment for common type of obesity.

### 2.2. Genome-Wide Linkage Studies

Genome-wide linkage studies are hypothesis generating and, through surveying the whole genome, aim to identify new, unanticipated genetic variants associated with a disease or trait of interest. Genome-wide linkage studies rely on the relatedness of study participants and test whether certain chromosomal regions cosegregate with a disease or trait across generations [17]. A whole-genome linkage survey requires 400–600 highly polymorphic markers, genotyped at 10 cM intervals. Thus, genome-wide linkage studies have a rather coarse resolution and typically identify broad intervals that require follow-up genotyping to pinpoint the genes that underlie the linkage signal. Since the first genome-wide linkage study was published in 1997 [34], the number of chromosomal loci linked to obesity-related traits has grown exponentially. The latest Human Obesity Gene Map update reported 253 loci from 61 genome-wide linkage scans, of which 15 loci have been replicated in at least three studies [29]. Yet, none of these replicated loci could be narrowed down sufficiently to pinpoint the genes or variants that underlie the linkage signal. Despite substantial power, a meta-analysis of 37 genome-wide linkage studies with data on >31,000 individuals from 10,000 families of European origin could not locate a single obesity or BMI locus with convincing evidence [35]. This meta-analysis indicates that genome-wide linkage analysis might not be an effective approach for identifying genetic variants for common obesity.

### 2.3. Genome-Wide Association Studies

Genome-wide association study is an approach used in genetics research to look for associations between many (typically hundreds of thousands) of specific genetic variations (most commonly, single-nucleotide polymorphisms) and particular diseases or traits. Similar to genome-wide linkage, the genome-wide association approach interrogates the entire genome, unconstrained by prior assumptions. Genome-wide association studies (GWAS) screen the whole genome at higher resolution levels than genome-wide linkage studies and are capable to narrow down the associated locus more accurately. The genome-wide association approach has effectively replaced genome-wide linkage approach for common disease [17, 22]. Three waves of large-scale high-density genome-wide association studies have led to a series of discoveries in the field of obesity genetics. Recent high-density multistage genome-wide association analyses have so far discovered ~30 loci consistently associated with BMI and obesity-related traits (Table 1). Recent excellent reviews have examined the implications of those loci to the development of human obesity [9, 22, 36]. The strongest signal remains the association with variants within FTO (the fat-mass and obesity-related gene) [37–39] (described in more detail below). Other signals near BDNF, SH2B1, and NEGR1 (all implicated in aspects of neuronal function) reinforce the view of obesity as a disorder of hypothalamic function [36].

It should be emphasized early on that whilst heritable factors are known to explain much of the individual variation in risk of obesity, genetic factors identified so far explain only a small proportion of this risk. The significance of this fact encourages researchers to identify new DNA variants influencing disease predisposition, in order to further understand the disease pathology, and to develop effective therapeutic and preventive strategies. It should be also highlighted that future strategies to identify this “missing heritability” include multiple methodological approaches, existing and novel. As a powerful and successful approach, surely genome-wide association studies will identify more sensitive genes or single nucleotide polymorphisms (SNPs) that influence BMI and the risk of obesity in the future.

## 3. Candidate Genes for Common Obesity

As listed in Table 1, candidate gene approach and large-scale high-density genome-wide association studies have identified new loci for common obesity. The discovery of these loci has initiated a series of experiments to explore the pathophysiological mechanisms that underlie obesity development, in particular to the fat mass and obesity-associated (FTO) gene. In 2007, SNPs within the FTO became the first to be associated reproducibly with human body mass. To date, of all identified loci, the genetic variation in FTO has the largest effect on obesity susceptibility [40]. FTO encodes a Fe(II)- and 2-oxoglutarate-dependent oxygenase putatively involved in DNA demethylation. Studies in rodents suggested that FTO mRNA is most abundant in the hypothalamic regions, which control energy homeostasis. The FTO protein shows wide expression patterns in central as well as peripheral regions [41]. Study also demonstrated that increased adipose tissue FTO mRNA was associated with increased BMI in healthy women [41]. FTO -null mice have reduced fat mass, and mice with FTO overexpression displayed increased energy intake and increased adiposity [42, 43]. Despite recent progress, the mechanism by which FTO influences human body mass remains elusive. Data from rodents suggested that FTO might affect neuropeptide Y (NPY, a feeding stimulation or orexigenic factor) expression in the hypothalamus, which in turn impacts feeding behavior [40]. Moreover, role of FTO in circadian rhythms has been proposed [44]. Aberrant circadian rhythms have been linked to metabolic disease and obesity [45].

Overall, multiple reports from genetics and epigenetic studies have shown compelling evidence that FTO is the strongest one of human obesity causing genes, even although the mechanisms by which FTO affect obesity are still not fully understood, the obesity risk alleles of FTO are gain of function. This also indicates that FTO is functionally involved in energy homeostasis by the regulation of energy balance, via food intake and/or energy expenditure.

Neuronal SH2B1 is essential for controlling energy and glucose homeostasis. Deletion of the SH2B1 gene resulted in metabolic disorders in SH2B1 knockout mice, including hyperlipidemia, leptin resistance, hyperphagia, obesity, hyperglycemia, insulin resistance, and glucose intolerance. Neuron-specific restoration of SH2B1*β* not only corrected the metabolic disorders in mice, but also improved JAK2-mediated leptin signaling and leptin regulation of orexigenic neuropeptide expression in the hypothalamus [46]. Moreover, neuron-specific overexpression of SH2B1 dose-dependently protected against high-fat diet-induced leptin resistance and obesity. These observations suggest that neuronal SH2B1 regulates energy balance, body weight, peripheral insulin sensitivity, and glucose homeostasis at least in part by enhancing hypothalamic leptin sensitivity. Variations close to or in the SH2B1 gene have been found to be associated with obesity in two large genome-wide association studies [47, 48]. SH2B1 deletions are associated with severe early-onset obesity [31]. Genetic differences among people can derive from lost or duplicated segments of chromosomes, called copy number variants (CNV). Variation in gene copy number can influence the activity of genes and accounts for a significant amount of genetic difference between people [49]. A recent work of Farooqi et al. analyzed the genomes of 300 obese children for missing or duplicated chromosome segments. Mutations in LEPR, POMC, and MC4R genes were previously excluded by direct nucleotide sequencing. The commonest CNV found in these patients was identified in five unrelated children harboring overlapping deletions on chromosome 16p11.2, which encompasses the gene SH2B1 [31]. The phenotype of the children with SH2B1-containing deletions is characterized by extreme hyperphagia and fasting insulin levels disproportionately elevated compared to age- and obesity-matched controls. Deletions on chromosome 16p11.2 have been reported in an adult population with severe obesity [50], further demonstrating the potential importance in SH2B1 gene variation and the development of human obesity.

Admixture mapping of 15,280 African Americans has identified three loci, 5q13,3, Xq13.1, and Xq25, that may harbor genetic variants associated with variations in BMI [51]. The 95% confidence interval (CI) for the chromosome 5 peak contains CART gene. Only a few studies report linkage of obesity with markers on the X chromosome. Three studies reported either suggestive or significant linkage of obesity to the q arm of chromosome X [52–54] and they all mapped to the Xq23-q24 region, which overlaps with the 95% CI of the highest admixture peak on chromosome X in this study. The 95% CI in this study contains one particular gene that may be a candidate for obesity susceptibility. The gene solute carrier family 6 member 14 (SLC6A14) is involved in serotonin synthesis and serotonergic receptor mechanisms that have been implicated in appetite control and body weight regulation [55]. Another potential candidate gene near the highest admixture peak is the cullin 4B (CUL4B) gene. CUL4B was recently identified as a causative gene for an X-linked mental retardation syndrome, which was associated with several clinical features, including central obesity [56].

## 4. Circadian Rhythms and the Pathogenesis of Obesity

As mentioned previously, the discovery of leptin represents a huge step in the study of obesity. Adipose tissue, until then considered as inert and metabolically inactive, is now regarded to be capable of producing proteins with autocrine, paracrine, and endocrine activity. Further discovery have shown that various adipokines, such as adiponectin, visfatin, resistin are produced in adipose tissue and involved in the pathophysiology of obesity [57]. Recent studies show that many genes in adipose tissue show circadian rhythmicity [58]. Microarrays carried out in animals and humans have shown that approximately 7 to 21% of active genes expressed in white and brown adipose tissue follow a daily rhythmic pattern [59]. Indeed, adipose tissue has been seen to express clock genes with a circadian rhythmicity, which is capable of triggering this tissue and even of modulating other genes, the so-called Clock control genes [60]. Circadian oscillators play an indispensable role in the coordination of physiological processes with the cyclic changes in the physical environment. A significant number of recent clinical and molecular studies suggest that circadian biology may play an important role in the regulation of adipose and other metabolic tissue functions and circadian dysfunction may be involved in the pathogenesis of obesity, type 2 diabetes, and the metabolic syndrome [61]. In mammals, the “master clock” driving circadian rhythm is located in the hypothalamic suprachiasmatic nucleus (SCN). The brain area contains neurons whose firing rates vary over an approximately 24-hour period and, in turn, coordinates the oscillation of “slave clocks” in other areas throughout the brain and periphery [62]. The molecular mechanisms underlying these oscillations were firstly described in Drosophila, but homologous genes have been identified across diverse species. The genes encoding the core clock mechanisms include circadian locomotor output cycles kaput (Clock), brain and muscle-Arnt-like 1 (Bmal1), Period-1 (Per1), Period-2 (Per2), Period-3 (Per3), Cryptochrome-1 (Cry1), and Cryptochrome2 (Cry2) [62].

Mouse Clock gene was identified in 1997 [63, 64]. Recent progress in our understanding of energy homeostasis may provide further information about how the Clock genetic variations confer obesity. A recent report from Turek et al., suggests that mutated Clock mice exhibited a near total loss of selective nocturnal feeding, consuming close to 50% of their daily food intake during the light phase. These mice are hyperphagic and develop obesity. The resultant obesity is associated with increases in blood glucose, cholesterol, and triglyceride levels, as observed in obese humans with the metabolic syndrome [65]. The obese phenotype of Clock mutant mice arises from loss of circadian rhythm is an important finding. This observation is compatible with the tight coupling between circadian clock and the patterns of feeding and physical activity. Master clock-controlled rhythms have been shown for blood glucose, glucose tolerance, and plasma leptin levels. Thus, it seems possible that other metabolism-related functions are also subject to regulation by the SCN or peripheral clocks [62]. Fluctuations in body weight have been associated with changes in day length in various species such as hamsters and sheep, suggesting a central role for the circadian clock in regulating body weight [62]. In humans, studies have demonstrated an increased incidence of obesity among shift workers [66]. Circadian clock plays a major role in determining body weight by influencing the expression and secretion of hormones. Leptin 24-hour levels were significantly lower in obese compared with nonobese adolescent girls [67], suggesting that blunted circadian variation may play a role in leptin resistance and obesity. Similarly to leptin, the rhythmic expression of resistin and adiponectin was greatly blunted in obese mice [67, 68]. In obese subjects, adiponectin levels were significantly lower than lean controls [69]. In rats, melatonin, a synchronizer of the SCN clock, decreased weight gain in response to high-fat diet and decreased plasma leptin levels. These effects were independent of total food consumption [70].

## 5. Role of Neuropeptides in Appetite Regulation and Obesity

The central nervous system plays a key part in regulating food intake through the brain-gut axis [30], with the hypothalamus acting as the central regulator, receiving both long- and short-term food intake and energy expenditure feedback from the periphery. The hypothalamus integrates these signals and acts through various downstream pathways to maintain energy balance. The hypothalamus is a master regulator of satiety, via production of pro-opiomelanocortin (POMC) and CART. POMC undergoes tissue-specific posttranslational cleavage, with the product depending on the endoproteases expressed in the tissue. In humans, leptin stimulates POMC conversion into *α*-MSH in the arcuate nucleus, which in turn binds to the MC4R, a key receptor involved in appetite control and energy homeostasis. Agouti-related peptide (AgRP) is an antagonist of MC4R. Mice overexpressing AgRP or MC4R knockout mice are hyperphagic and obese [71] and are insensitive to *α*-MSH. MC4R mutations have been found in about 5.8% of adults with severe childhood-onset obesity [72]. In another report it has been shown that resequencing MC4R resulted in the identification of low-frequency coding variants that explain approximately 2 to 3% of cases of severe obesity [73]. Common variants near MC4R are associated with fat mass, weight and risk of obesity [74]. POMC deficiency could also lead to obesity (due to lack of binding at MC4R), hypocortisolism (due to the lack of binding of ACTH to the MC2R in the adrenal gland), and alteration of pigment (due to lack of binding at MC1R in the skin). This syndrome is defined by severe early onset obesity, adrenal insufficiency, and red hair [75]. In rodent models of cancer [76] and renal failure [77], MC4R antagonists attenuate cachexia by maintaining appetite, lean body mass, and basal energy expenditure [78]. Thus, MC4R antagonists may be useful to treat cachexia [79] while MC4R agonists are being developed to treat obesity.

Another important satiety regulator in the hypothalamus is CART, which is coexpressed with POMC in arcuate neurons in animals and humans [80]. CART is a hypothalamic neuropeptide that transmits a physiological anorexigenic signal and is involved in appetite regulation. CART knockout mice have increased body weight compared with wild-type mice [81]. Genomic regions containing the CART gene have been linked to both BMI and serum leptin levels in a study of French Caucasian families [82]. SNPs in the 5′ upstream region have been reported to be associated with obesity in Japanese [83] and French [84]. Similar to POMC neurons, CART neurons are directly stimulated by leptin [18, 85]. CART neurons target areas throughout the hypothalamus and are associated with reinforcement and reward, sensory processing, and stress and endocrine regulation. CART expression also exhibits food-dependent diurnal rhythm in blood and brain [86]. Animals deprived of food have decreased the expression of CART mRNA. Intracerebroventricular administration of CART in rats inhibits normal and starvation-induced feeding, as well as blocking the NPY feeding response [18, 85, 87]. CART may be useful to combat obesity. However, clinical trial utilizing CART agonists or antagonists for weight regulation is still lacking. CART receptor has not been cloned. However, the first interaction partner for CART was recently identified by yeast two-hybrid system. Succinate dehydrogenase, a unique mitochondrial protein of both the Krebs TCA cycle and complex II of the electron transport chain, is a crucial enzyme for intermediary metabolism and energy production [88]. Thus, the biochemical and physiological linkage between CART and energy homeostasis has been firmly established.

Various peripheral neuropeptides regulate appetite and influence the development of obesity. Of these, ghrelin, or growth hormone- (GH-) releasing peptide, is the only known circulating orexigen, or appetite stimulant. Ghrelin is gut hormone. Ghrelin acts as a meal initiator as ghrelin levels rise prior to meals, and falls quickly after ingestion of nutrients. The ghrelin receptor, GH secretagogue receptor type 1a (GHS-R1a), is a G-protein coupled receptor. Exogenous ghrelin administration affects glucose homeostasis, gut motility, pancreatic exocrine secretion, cardiovascular function, immunity, and inflammation [89]. Administration of ghrelin to obese and lean human subjects leads to increased food intake, in part by stimulating the production of NPY and AgRP in the arcuate nucleus. Ghrelin may also alter energy balance by stimulating adipogenesis, inhibiting apoptosis, transitioning from fatty acid oxidation to glycolysis for energy expenditure, and inhibiting sympathetic nervous system activity. Future studies may examine ghrelin antagonists as a therapeutic option for obesity [90, 91]. The biological activities of ghrelin require octanoylation of the peptide on Ser3, a posttranslational modification that is catalyzed by the enzyme ghrelin O-acyltransferase (GOAT). GO-Coa-Tat, a peptide-based analog that antagonizes GOAT inhibits GOAT in vitro, in cultured cells, and in mice. Intraperitoneal administration of GO-Coa-Tat improves glucose tolerance and reduces weight gain in wild-type mice but not in ghrelin knockout mice [92], suggesting its beneficial metabolic effects are due to specific GOAT inhibition. In addition to ghrelin, many peripheral neuropeptides may also be associated with satiety. The list of satiety peripheral hormones is extensive. Important peripheral neuropeptides include cholecystokinin (CCK), peptide YY (PYY3-36), pancreatic polypeptide (PP), incretins, glucagon-like peptide-1 (GLP-1), glucose-dependent insulinotropic polypeptide (GIP), amylin, and bombesin. These peripheral hormones regulate gastrointestinal functions such as motility, secretion, and absorption and provide feedback to the central nervous system on availability of nutrients and may play an important role in regulating energy intake.

## 6. Genes and Biochemical Pathways in Peripheral Organs/Tissues Influence Energy Homeostasis

New strategies for gene identification have emerged. One source of novel intermediate phenotype with the utilization of microarray technology has provided valuable information on mRNA expression from thousands of genes in tissues of interest. cDNA microarray analysis for the study of obesity was first reported by Soukas et al. in 2000, using approximately 6,500 murine genes in pairs of adipose tissues in *ob/ob *mice and wild-type lean mice [93]. This was followed by many subsequent studies. More than 30 microarray approaches have been exploited in assessing the changes in gene expression in the adipose tissue, liver, hypothalamus, skeletal muscles, small intestines, and kidneys of lean and obese animals and human subjects. The data obtained from these genomic investigations have provided a wealth of information about obesity-specific gene profiles. The overexpression of genes related to inflammation, immune response, adhesion molecules, and lipid metabolism is a major characteristic of white adipose tissue, while the overexpression of the genes related to lipid metabolism, adipocyte differentiation, defense, and stress responses, is noticeable in the nonalcoholic fatty liver of obese rodents [94]. The hepatic-gene expression suggests that in obese rodents, the livers are supplied with large amounts of fatty acids under conditions associated with obesity either through increased fatty acid biosynthesis or through decreased fatty acid oxidation, which may lead to increased mitochondrial respiratory activity.

Brain-derived neurotrophic factor, BDNF, with its receptor Trk B has been shown to regulate neuronal development and plasticity and it is well established that BDNF plays a role in the hypothalamic pathway that controls body weight and energy homeostasis. Very interestingly, recent evidence identifies BDNF as a player not only in central metabolism, but also in regulating energy metabolism in peripheral organs, such as pancreatic islets and skeletal muscle [95, 96]. Similarly, peptide CART is also expressed in peripheral organs, including adrenal glands and pancreatic islets [21]. This feature supports the idea that neurotrophic factors may act via multiple pathways in controlling energy balance, disruption of the normal function and regulation in any levels (center/global, local) may eventually affect the development of weight disorders.

## 7. Oxidative Stress and Cognitive Deficits in Obesity

Oxidative stress is a key factor that is involved in many chronic neurodegenerative diseases, including the main form of dementia, Alzheimer's disease [97, 98]. Oxidative stress is also viewed as a primary mechanism linking obesity and metabolic disorders [99]. Therefore, oxidative stress may be a common mechanism for these complex diseases. Increased evidence indicates that there is an association between obesity and nervous system disorders (cognitive deficits, neurodevelopmental disorders, neurodegenerative diseases, and psychiatric disorders), including dementia and major depression. The conditions of chronic obesity and overweight status are risk factors for lower cognitive performance, cognitive decline, cognitive deficit, and dementia; on the other hand, lower cognitive performance early in life itself may be a risk factor for an increase in body weight over time [100]. Childhood overweight and obesity and their psychosocial and cognitive consequences have been investigated in a nationally representative sample of children. The association between BMI and academic performance was not significant after adjusting for parental/familial characteristics. However, the associations between BMI and cognitive functioning remained significant, and severe overweight correlates with lower cognitive functioning [101].

Notably, recent investigations report a new highly penetrant form of obesity (or underweight) due to deletions (or reciprocal duplications) on chromosome 16p11.2, that is often associated with hyperphagia and intellectual disabilities [50, 102], indicating a pathological connection between weight disorders and nervous system diseases in genomic level. The reciprocal impact of these 16p11.2 copy-number variants also indicates that severe obesity and being underweight could have mirror etiologies, possibly through contrasting effects on energy balance. The precise mechanisms underlining the phenotypes/disorders are currently unclear, but they are worth further exploring. Interestingly some genes have been shown to be involved in both obesity and CNS disorders, such as neurotrophin BDNF and neurotransmitter CART [21, 103, 104].

## 8. The Critical Role of Lifestyle on Obesity

Genetic alterations including mutations, deletions, and SNPs only explain a small part of obesity, but the total number of obesity has been significantly increasing in recent decades. Therefore, it may be more importantly that lifestyle changes in the last decades have greatly contributed to the current obesity trends. It is widely known that diet and dietary habits as well as less or lack of exercises do play a role in the development of obesity.

Dietary factors may modify body weight as well as obesity. There is even controversy concerning the role of sugar in the epidemics of obesity and metabolic syndrome, there is less controversy concerning the effects of fructose on obesity and metabolic syndrome. Increasing evidence that sugar consumption promotes development of an unfavorable lipid profile is strong and suggests that the upper added sugar consumption limit of 25% energy or less, suggested in the Report of the Dietary Guidelines Advisory Committee on the Dietary Guidelines for Americans 2010, may merit reevaluation [105].

On the other hand, evidence from a variety of animal models including rodents and nonhuman primates indicates that exposure to maternal high-fat diet (HFD) consumption programs offspring for increased risk of adult obesity [106]. Hyperphagia and increased preference for fatty and sugary foods are implicated as mechanisms for the increased obesity risk. Using a nonhuman primate model of diet-induced obesity, maternal HFD consumption caused perturbations in the central serotonergic system of fetal offspring. In addition, female infants from HFD-fed mothers exhibited increased anxiety in response to threatening novel objects. These findings demonstrate that exposure to maternal HFD consumption during gestation, independent of obesity, increases the risk of developing behavioral disorders such as anxiety [107]. As maternal HFD consumption and obesity are common and rapidly increasing, it was speculated that future generations will be at increased risk for both metabolic and mental health disorders. Reduced physical activity and/or increasing sedentary behavior, such as television viewing, is implicated in the etiology of obesity. Definitive evidence of a role for reduced physical activity and energy expenditure in the causation of obesity is lacking. However, there is stronger evidence that targeting activity and/or inactivity might be effective in pediatric obesity treatment [108].

As mentioned early, BDNF has been shown to regulate neuronal development and plasticity and plays a role in learning and memory. Low levels of BDNF are found in patients with neurodegenerative diseases, including Alzheimer's disease and major depression. In addition, BDNF levels are low in obesity and independently so in patients with type 2 diabetes. Interestingly it has been shown that exercise increases BDNF levels not only in the brain and in plasma, but in skeletal muscle as well [109]. BDNF mRNA and protein expression was increased in muscle cells that were electrically stimulated, and BDNF increased phosphorylation of AMP-activated protein kinase (AMPK) and acetyl coenzyme A carboxylase-beta (ACCbeta) and enhanced fatty oxidation both in vitro and ex vivo. Thus, BDNF appears to play a role both in neurobiology and in central as well as peripheral metabolism. The finding of low BDNF levels both in neurodegenerative diseases and in type 2 diabetes may explain the clustering of these diseases. Therefore BDNF is likely to mediate some of the beneficial effects of exercise with regard to protection against dementia, obesity, and type 2 diabetes [109].

## 9. Summary and Future Directions

Obesity is influenced by a complex interaction between the environment, genetic predisposition, and human behavior. It is associated with an increased risk of numerous chronic diseases. It is also associated with an increased risk of death in populations of European ancestry and black women [110]. As a result, obesity epidemic exerts a heavy toll on the economy with its massive health care costs. There is growing evidence that genetic predisposition confers obesity. Nevertheless, despite the enormous success of genetic studies, there are still important gaps in this knowledge. The established loci in combination can only explain <2% of interindividual variation in BMI [111]. Given that the heritability of BMI is estimated at 40–70%, it is reasonable to assume that many more susceptibility loci remain to be uncovered. To this task, powerful genome-wide association studies and other genome-level approaches are expected to be utilized widely, including large-scale genome-wide resequencing and global level of corporation.

Of interest is that many of the currently identified obesity susceptibility loci locate near genes that are highly expressed in the brain especially hypothalamus, favoring a role for the nervous system in body weight control. Latest reports have shown the association between obesity and CNS disorders, such as cognitive defects. Central and peripheral neuropeptides regulate appetite and energy expenditure and may play an important role in the development of obesity. Recent studies also suggest that obesity may be associated with a fault in the circadian system. Adipose tissue is capable to produce protein with autocrine, paracrine, and endocrine activity. Adipokines such as adiponectin, visfatin, and resistin are involved in the physiology of obesity and those genes show circadian rhythmicity. Furthermore, obesity-specific gene expression profiling in various peripheral tissues may help in understanding the pathogenic mechanisms of obesity and its associated metabolic diseases.

Recent advances in identifying genetic risk factors for obesity have contribution to understanding disease pathology, which may facilitate the formation of new therapeutic strategies, including personalized medicine.

## Figures and Tables

**Table 1:** Overview of main obesity-susceptibility loci identified through candidate gene and genome-wide association studies.

Chromosomal location	Gene symbol	Gene name
1p31.1	NEGR1	Neuronal growth regulator 1
1q41	LYPLA1	Lysophospholipase-like 1
1q25.2	SEC16B	SEC 16 homologue B
	RASAL2	RAS protein activator like 2
2p25.3	TMEN18	Transmembrane protein 18
2q14.1	INSIG2	Insulin-induced gene 2
3q27	ETV5	Ets variant gene 5
4p13	GNPDA2	Glucosamine-6-phosphate deaminase 2
5q13.3	CART	Cocaine-and amphetamine-regulated transcript
5q15-q21	PCSK1	Prohormone convertase 1/3
6p12	TFAP2B	Transcription factor AP-2*β*
6p22.2-p21.3	PRL	Prolactin
6q14-q15	CNR1	Endocannabinoid receptor 1
8p12-p11.2	ADRB3	Adrenergic *β*3 receptor
8p23.1	MSRA	Methionine sulfoxide reductase A
10p12	PTER	Phosphotriesterase related
11p11.2	MTCH2	Mitochondrial carrier homologue 2
11p13	BDNF	Brain-derived neurotrophic factor
12q13	BCDIN3D	BCDIN3-domain-containing
	FAIM2	FAS apoptotic inhibitory molecule 2
14q31	NRXN3	Neurexin 3
16p11.2	SH2B1	SH2B adaptor protein 1
	ATP2A1	ATPase, Ca^2+^ transporting, cardiac muscle, fast twitch 1
16q12.2	FTO	Fat mass and obesity-associated gene
16q22-q23	MAF v	Maf musculoaponeurotic fibrosarcoma oncogene homologue
18q11-q12	NPC1	Niemann-Pick disease, type C1
18q22	MC4R	Melanocortin 4 receptor
19q13.11	KCTD15	Potassium channel tetramerisation domain containing 15
19q31	CHST8	Carbohydrate (N-acetylgalactosamine 4-0) sulfotransferase 8
Xq23-24	SLC6A14	Solute carrier family 6 member 14
	CUL4B	Cullin 4B
